# N-Acetylcysteine Priming Alleviates the Transplanting Injury of Machine-Transplanted Rice by Comprehensively Promoting Antioxidant and Photosynthetic Systems

**DOI:** 10.3390/plants11101311

**Published:** 2022-05-15

**Authors:** Wenjun He, Qiuyi Zhong, Bin He, Boyang Wu, Atta Mohi Ud Din, Jielyv Han, Yanfeng Ding, Zhenghui Liu, Weiwei Li, Yu Jiang, Ganghua Li

**Affiliations:** 1 China-Kenya Belt and Road Joint Laboratory on Crop Molecular Biology, Nanjing 210095, China; 2019201028@njau.edu.cn (W.H.); 100002111@gxust.edu.cn (Q.Z.); 2020201025@stu.njau.edu.cn (B.H.); 2021801223@stu.njau.edu.cn (B.W.); attajutt82@yahoo.com (A.M.U.D.); jielyu.han@wur.nl (J.H.); dingyf@njau.edu.cn (Y.D.); liuzh@njau.edu.cn (Z.L.); li1990@njau.edu.cn (W.L.); yujiang@njau.edu.cn (Y.J.); 2 Key Laboratory of Crop Physiology Ecology and Production Management, Ministry of Agriculture, Nanjing Agricultural University, Nanjing 210095, China; 3 Jiangsu Collaborative Innovation Center for Modern Crop Production, Nanjing Agricultural University, Nanjing 210095, China; 4 Library, Guangxi University of Science and Technology, Liuzhou 545005, China; 5 National Engineering and Technology Center for Information Agriculture, Nanjing 210095, China

**Keywords:** antioxidant, machine-transplanted rice, N-acetylcysteine, photosynthetic, transplanting injury

## Abstract

The stress of transplanting injury adversely affects rice growth and productivity worldwide. N-acetylcysteine (NAC), the precursor of glutathione, is a potent ROS scavenger with powerful antioxidant activity. Previous studies on the application of NAC in plants mainly focused on alleviating the stress of heavy metals, UV-B, herbicides, etc. However, the role of NAC in alleviating transplanting injury is still not clear. A barrel experiment was carried out to explain the mechanism of NAC regulating the transplanting injury to machine-transplanted rice during the recovery stage. The results showed that NAC priming shortened the time of initiation of tillering and increased the tiller numbers within 3 weeks after transplanting. In addition, NAC priming increased the chlorophyll content, net photosynthetic rate, and sucrose content, thereby improving the dry weight at the recovery stage, especially root dry weight. At the same time, NAC priming significantly increased the activity of ascorbate peroxidase (APX), glutathione reductase (GR), catalase (CAT), and superoxide dismutase (SOD). In addition, it also regulated flavonoids and total phenols contents to reduce hydrogen peroxide (H_2_O_2_) and malondialdehyde (MDA) contents, especially at the initial days after transplanting. These results suggest that NAC priming improves the tolerance of rice seedlings against transplanting injury by enhancing photosynthesis and antioxidant systems at initial days after transplanting, thereby promoting the accumulation of dry matter and tillering for higher yield returns.

## 1. Introduction

Rice (*Oryza sativa* L.), one of the most widely cultivated staple foods, is consumed by more than 50% of the population of the world [1]. China is amongst the largest rice-producing countries and contributes about 35% of the global rice production [2]. Considering the high consumption of rice and the burgeoning population, improvement in rice production technology and yield is very necessary to meet the growing demand of food in the world [3]. However, high-paced urbanization and quests for a better lifestyle have encouraged the labor to shift towards cities, which has caused a labor shortage in rural areas. Therefore, rice mechanization needs to be improved to tackle such problems of the modern world. 

The mechanization of rice nursery transplantation methods such as mechanical carpet-transplanting has reduced the requirement of labor, and is being used widely in China. However, the severe mechanical damage caused to seedlings during such transplantation still remains a point of concern for rice researchers [4]. Previous studies have shown that physical or mechanical injuries might cause a drastic hindrance in the growth and yield of crop plants [5]. Such wounding causes cell death and water loss at the injured sites, and damaged plants are also easily prone to pathogenic attacks which could intensify the stress through these wounds [6,7,8]. Consequently, it induces over-production of reactive oxygen species (ROS) in plant [9], which then triggers the lipid peroxidation of cellular membranes and causes significant damage to photosynthetic machinery, including pigments, proteins, and lipids [10,11]. Damaged plants will regulate photosynthetic capacity and the partitioning of resources through multiple signaling factors such as jasmonic acid (JA), ABA, and ROS to defend against stress [12,13,14]. However, much energy is needed for the process of defense, which may compete with growth and reproduction [15]. In addition, root injuries during transplantation impede the transportation of amino acids and hormones towards leaves that are synthesized in roots and play important role plant defense responses [16]. Similarly, studies also reported that root injury might impair the absorption capacity of Zn and other important nutrients from soil [17]. Therefore, transplanting injury inhibits the growth of rice seedlings, resulting in a longer recovery stage. Considering these issues, it is essential to effectively reduce the adverse impacts caused by the transplanting injury. 

A number of studies suggest that chemical priming is a promising and potential field in plant stress management [18]. N-Acetyl-L-cysteine (NAC, C_5_H_9_NO_3_S) is a thiol compound derived from cysteine amino acid, which is widely used as a chelating agent, antioxidant, and active oxygen scavenger in medicine [19]. Recent plant studies have shown that exogenous NAC reduces adverse effects resulting from biotic and abiotic stresses [20,21,22]. NAC has been reported to reduce the bacterial population in the sweet orange ‘*Pera*’ caused by the Xylella fastidiosa [20]. It also possess important role in alleviating the effects of UV-B stress in *Anabeana* sp. and *Glycine max var. Hood* [23,24]. Treatment with NAC is also effective in *Hordeum vulgare* L. and *Solanum nigrum* L. [21,22] to alleviate Cd stress, and in the aquatic macrophyte *Callitriche obtusangula* exposed to herbicides [25]. In addition, NAC improves some physiological functions such as chlorophyll content, chlorophyll fluorescence, and photosynthesis rate to enhance crop production.

Considering the roles of NAC against abiotic stress in plants and the negative impact of transplanting injury on rice, it was hypothesized that NAC priming could alleviate transplanting injury. Therefore, our study investigated the comprehensive regulation of NAC priming on antioxidant systems and photosynthetic capacity of machine-transplanted rice after transplanting. Additionally, the physiological mechanism that NAC priming mitigated the transplanting injury of machine-transplanted rice is also discussed in this paper.

## 2. Results

### 2.1. Phenotype, Yield, and Yield Components

**Figure 1:**
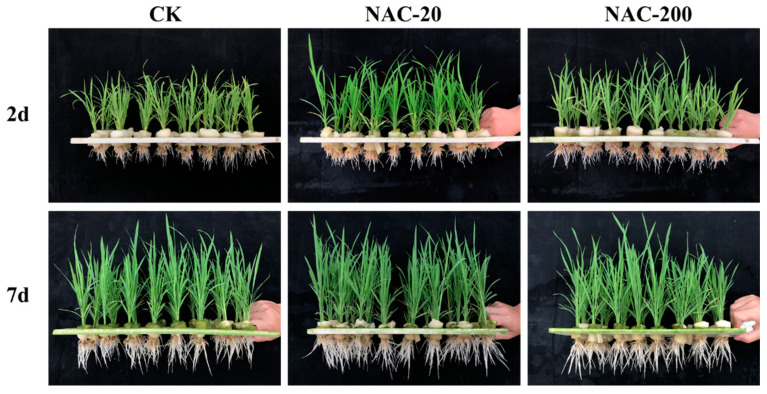
Effects of different concentrations of NAC on seedling growth at the 2 and 7 days after transplanting. CK, 0 μm; NAC-20, 20 μm; NAC-200, 200 μm.

The phenotype of rice plants grown in hydroponic culture clearly showed a positive effect of NAC priming, particularly NAC-20, on the growth of rice seedlings (Figure 1). Consistently, compared with CK,NAC-20 significantly increased the number of panicles, the grain filling rate, and the yield of rice (Table 1). Both NAC-20 and NAC-200 had higher yields than CK (11.2% and 8.1%, respectively), suggesting the positive response of rice seedlings towards NAC priming.

### 2.2. Tiller Dynamics

**Figure 2:**
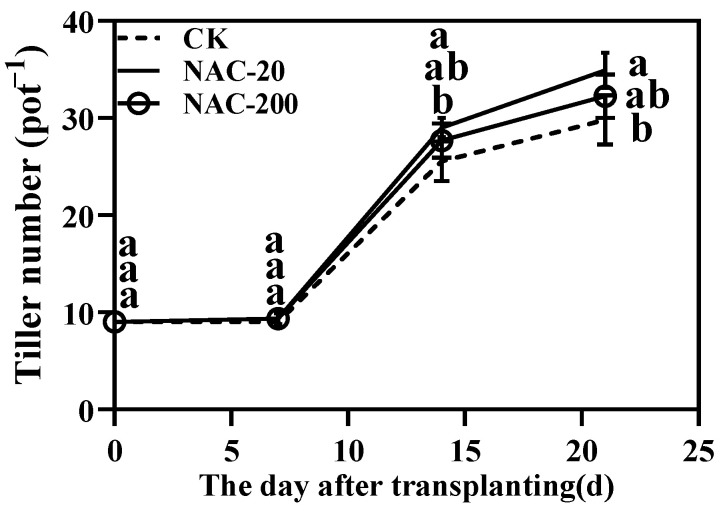
Effects of different concentrations of NAC on the tiller numbers within 3 weeks after transplanting. CK, 0 μm; NAC-20, 20 μm; NAC-200, 200 μm. Data are means ± SD (*n* = 3). The different letters following average values indicate significant difference (Duncan, *p* < 0.05).

NAC increased the tiller numbers within 3 weeks after transplanting (Figure 2). During the second week, a sharp increase in tiller number was noticed in all three treatments, but NAC-treated rice seedlings showed a significantly higher tiller number than CK. Importantly, the highest tiller number was always noticed (NAC-20) after the first week of transplanting. The NAC-20 had 17.1% and 8.4% higher tiller numbers than CK and NAC-200, respectively. 

### 2.3. Plant Growth Parameters

**Figure 3:**
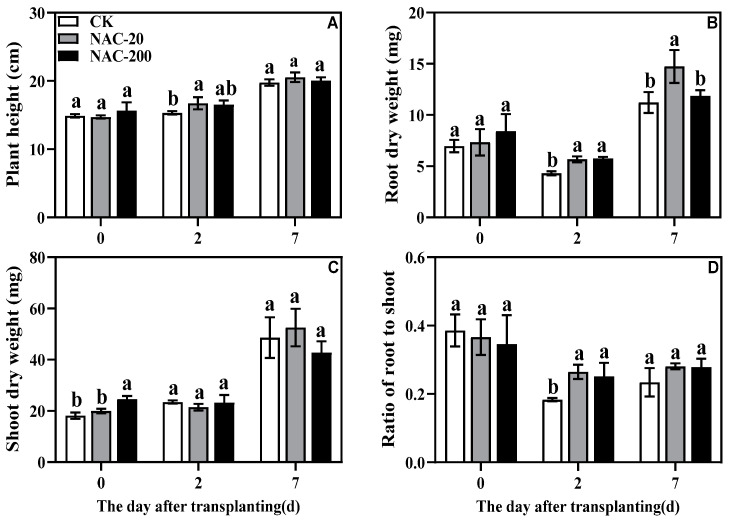
Effects of different concentrations of NAC on plant height (**A**), root dry weight (**B**), shoot dry weight (**C**), and ratio of root to shoot (**D**). CK, 0 μm; NAC-20, 20 μm; NAC-200, 200 μm. Data are means ± SD (*n* = 3). The different letters following average values indicate significant difference (Duncan, *p* < 0.05).

The NAC treatments showed dynamic response on the growth parameters of rice seedlings during the first week of transplantation (Figure 3). Compared to CK, the NAC treatment significantly improved plant height, shoot dry weight, and root dry weight, which was more obvious within the 2 days after transplanting. However, there was no significant difference in most growth parameters at 7 days after transplanting, suggesting that NAC mainly helped to regulate the initial growth during transplantation shock. In addition, transplanting decreased the root-to-shoot ratio in all treatments, but NAC-treated seedlings retained a significantly higher root-to-shoot ratio than CK.NAC-20 showed more obvious impact on growth parameters at 2 or 7 days after transplanting. 

### 2.4. Chloroplast Pigment Content

**Figure 4:**
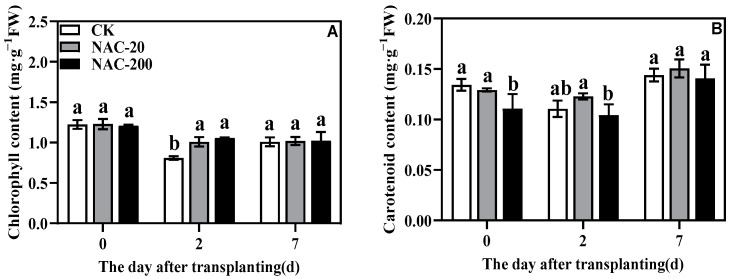
Effects of different concentrations of NAC on chlorophyll (**A**) and carotenoid (**B**) content. CK, 0 μm; NAC-20, 20 μm; NAC-200, 200 μm. Data are means ± SD (*n* = 3). The different letters following average values indicate significant difference (Duncan, *p* < 0.05).

Initially, at 0 days, there was no obvious different in chlorophyll content, but it declined markedly after the 2 days of transplanting, more obviously in CK (Figure 4A). At this stage, both NAC treatments showed significantly higher chlorophyll content than CK. A similar trend was noticed in carotenoids content at 2 days after transplanting (Figure 4B). However, within NAC treatments, NAC-20 showed higher carotenoids content than NAC-200. 

### 2.5. Gas Exchange Parameters

**Figure 5:**
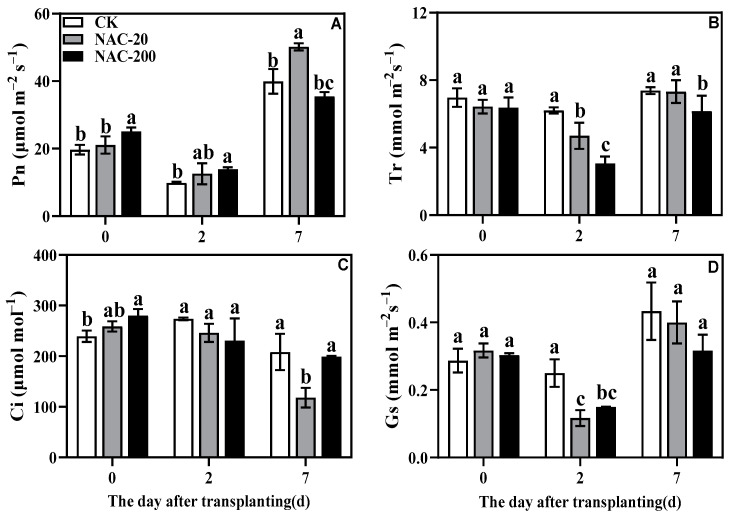
Effects of different concentrations of NAC on Pn (**A**), Tr (**B**), Ci (**C**), and Gs (**D**). CK, 0 μm; NAC-20, 20 μm; NAC-200, 200 μm. Data are means ± SD (*n* = 3). The different letters following average values indicate significant difference (Duncan, *p* < 0.05).

The application of NAC had a significant effect on Pn on each day (Figure 5A). The Pn of NAC-200 was significantly higher than that of CK at 0 and 2 days, while the Pn of NAC-20 was significantly higher than that of CK and NAC-200 at 7 days. Compared with CK,NAC-20 and NAC-200 significantly reduced Tr and Ci at 2 and 7 days (Figure 5B,D). NAC-200 also significantly increased Ci at 0 days (Figure 5C).

### 2.6. Sucrose Content

**Figure 6:**
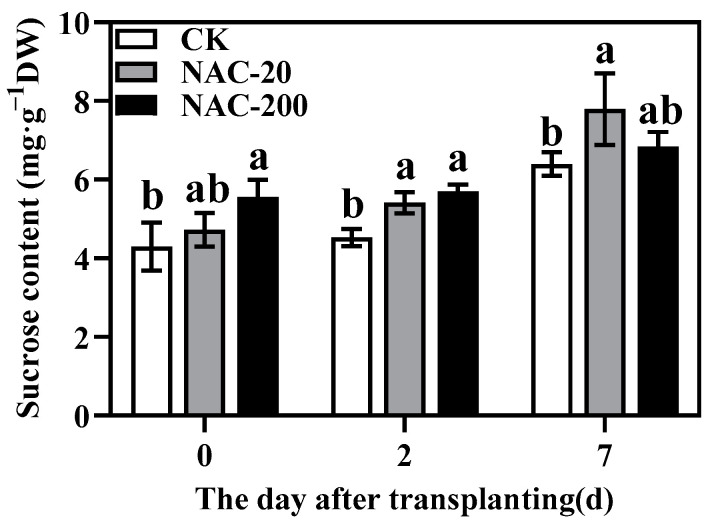
Effects of different concentrations of NAC on sucrose content. CK, 0 μm; NAC-20, 20 μm; NAC-200, 200 μm. Data are means ± SD (*n* = 3). The different letters following average values indicate significant difference (Duncan, *p* < 0.05).

Sucrose content increased continuously after transplantation of rice seedlings in all treatments, and NAC treated seedlings always showed higher sucrose content than CK (Figure 6). Importantly, after 7 days, the highest sucrose accumulation was observed in the leaves of NAC-20 plants. 

### 2.7. Antioxidant Enzyme Activity

**Figure 7:**
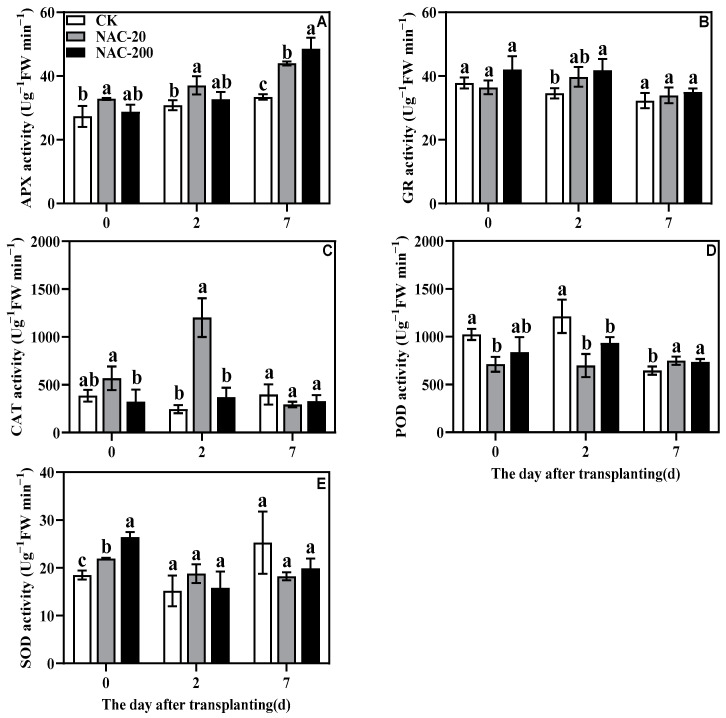
Effects of different concentrations of NAC on APX (**A**), GR (**B**), CAT (**C**), POD (**D**), and SOD (**E**). CK, 0 μm; NAC-20, 20 μm; NAC-200, 200 μm. Data are means ± SD (*n* = 3). The different letters following average values indicate significant difference (Duncan, *p* < 0.05).

Compared to CK, NAC treatment significantly increased the activities of the major antioxidant enzymes except POD, particularly within 2 days of transplantation (Figure 7). For instance, the NAC-20 seedlings showed the highest activity for SOD, CAT, and APX, suggesting that these enzymes are more responsive towards NAC treatment. 

### 2.8. Non-Enzymatic Antioxidant Content

**Figure 8:**
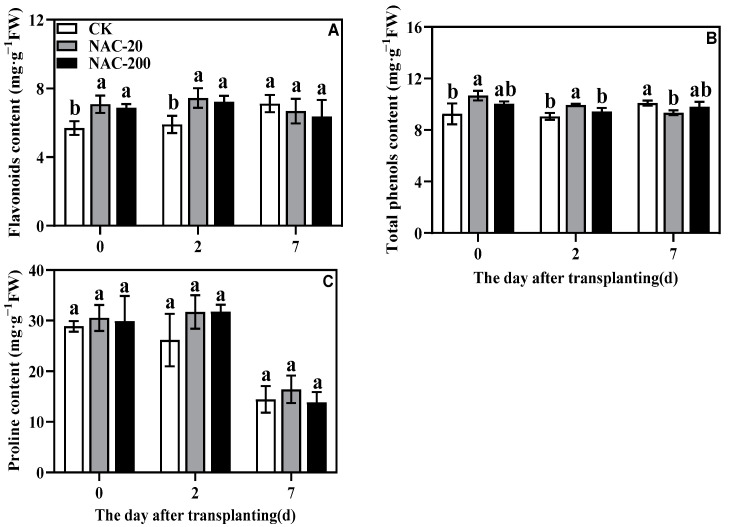
Effects of different concentrations of NAC on flavonoids (**A**), total phenols (**B**), and proline (**C**) content. CK, 0 μm; NAC-20, 20 μm; NAC-200, 200 μm. Data are means ± SD (*n* = 3). The different letters following average values indicate significant difference (Duncan, *p* < 0.05).

NAC treatment increased the flavonoid and total phenol contents at 0 and 2 days, with NAC-20 showing the highest content (Figure 8A,B). Proline content did not show any significant difference between CK and NAC-treated seedlings; but the highest proline accumulation was always noticed in NAC-20 plants (Figure 8B). 

### 2.9. H_2_O_2_ and MDA Content

**Figure 9:**
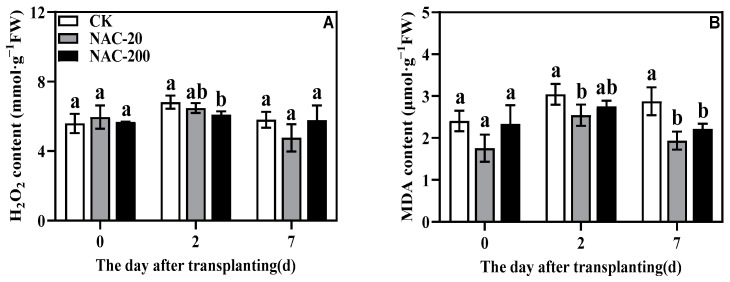
Effects of different concentrations of NAC on H_2_O_2_ (**A**) and MDA (**B**) content. CK, 0 μm; NAC-20, 20 μm; NAC-200, 200 μm. Data are means ± SD (*n* = 3). The different letters following average values indicate significant difference (Duncan, *p* < 0.05).

The H_2_O_2_ and MDA content of each treatment first increased and then decreased (Figure 9A,B). However, NAC treated seedlings showed lower transplantation stress, as noticed by their significantly lower H_2_O_2_ and MDA content than CK. Importantly, at 7 days, NAC-20 showed the lowest H_2_O_2_ and MDA contents, compared to NAC-200 and CK. 

## 3. Discussion

In recent times, mechanical transplanting methods have been replacing the conventional methods of rice cultivation, due to labor shortage in rural areas and increases in labor costs [26]. Among mechanical transplanting methods, carpet seeding methods have been popularized in many rice cultivation regions of China. However, the plant and root injury during machine transplanting results in the production of weak young seedlings [27]. A wide range of studies have proven the viability of exogenous application of materials that are already involved in several endogenous metabolic process of the organism, i.e., melatonin, polyamines, phytohormones, etc. against abiotic stresses [28]. Similarly, NAC has been utilized in pharmaceutical drugs and nutritional supplement against various diseases due to its role as a free radical scavenger and chelating agent [29]. In addition, some studies in plants also showed its positive effects against various abiotic and biotic stresses [20,21]. Therefore, to further elaborate the positive role of NAC against transplanting injury, we carried out this study with two different concentrations of NAC. Our results show that NAC priming is effective in alleviating the transplanting shock during early days after transplanting. It enhanced the antioxidant capacity and promoted the growth and tillering of rice seedlings compared to control plants, ultimately resulting in significantly a higher yield for NAC-treated plants than CK.

### 3.1. The Concentration of 20 μM NAC Priming Is More Effective in Promoting Tillering and Yield of Machine-Transplanted Rice under Transplanting Injury

Transplanting injury has a considerable impact on rice growth and generally delays the development of rice [30]. Recent studies have shown that seed treatment with exogenous materials could improve the tolerance of rice against transplanting injury and chilling stress [31]. The exogenous application of NAC in wheat plants protected the roots against heavy metal stress and improved the growth and biomass of wheat plants [29]. Consistently, the NAC-primed seedlings in our study showed significantly higher yield components than CK. Although previous studies did not show any harmful effect of NAC, even at high concentrations, different concentrations may induce variable responses in different species. For instance, application of 200 µM NAC effectively protected the barley roots and leaves against Cd stress [21], but for Solanum nigrum, the concentration of 500 µM was effective against the same heavy metal stress [22]. Similarly, in our study, 20 µM of NAC (NAC-20) appeared to be more effective in terms of higher numbers of tillers (Figure 2) and grain yield (Table 1), and biomass accumulation. Altogether, these results suggest that NAC priming could be adopted as a promising strategy to reduce the negative influence of transplanting injury and promote tiller growth on machine-transplanted rice.

### 3.2. NAC Priming Improves the Photosynthetic Capacity and Promotes the Accumulation of Sucrose and Dry Matter of Machine-Transplanted Rice under Transplanting Injury

Mechanized transplanting injury can break the balance between water absorption and transpiration, and affect various metabolic processes such as photosynthesis [32]. The main photosynthetic pigments are chlorophyll a, chlorophyll b, and carotenoids, which are an indispensable part of photosynthesis [33]. Previous studies have shown that the chlorophyll content [34], Pn, and light saturation point of plants are reduced under stress [35]. Zn-induced chlorophyll (Chl) content and Chl fluorescence (F_m_) of corn were increased by the NAC treatment [36], which increased the Pn rate and PAR [37]. This study showed that transplanting injury reduced chlorophyll content, Pn, Tr, and Gs at the early stage of injury. Compared with CK, however, NAC treatment had higher chlorophyll and Pn (Figure 4 and Figure 5), which maintained photosynthesis of plants under transplanting injury. Therefore, NAC treatments alleviated the inhibition of transplanting injury on rice growth, which was mainly manifested in increased sucrose content and root dry weight (Figure 3 and Figure 6). Some studies suggested that applications of NAC lead to increases in the sucrose, fructose, and glucose contents of the root [38]. It is considered that root growth is most critical for post-transplanting growth of plants [39]. In conclusion, NAC alleviates the effect of transplanting injury on photosynthesis and promotes dry matter accumulation to improve the growth of machine-transplanted rice after transplantation.

### 3.3. NAC Priming Improves the Antioxidant Capacity to Reduce Transplanting Injury of Machine-Transplanted Rice

It has been well-established that environmental stresses and physical injuries induce oxidative damage and results in lipid peroxidation [31]. As a reactive oxygen species, H_2_O_2_ is an especially important signaling molecule; however, its burst production under stressful conditions could impose severe oxidative stress and cause cellular damage [40]. From this perspective, MDA is one of the well-known markers for possible membrane damage due to lipid peroxidation. In the present study, the initial increase in H_2_O_2_ accumulation and lipid peroxidation (MDA) within 2 days of transplanting indicates the transplanting-induced oxidative damage in rice seedlings. However, compared to control, the significantly lower MDA and H_2_O_2_ content in NAC treatments suggests that exogenous NAC is involved in mitigating the impact of transplanting shock during the early days of rice transplanting (Figure 9). Previous studies with different abiotic stresses have shown that NAC application is very effective in regulating the antioxidant system and direct scavenging of excessive ROS [25,38]. Therefore, to further verify the role of NAC against transplanting injury, we also compared the response of antioxidants in CK and NAC plants. Consistent with the lower oxidative damage in NAC treated seedlings, the activities of major antioxidant enzymes SOD, APX, CAT, and GR were found significantly higher in them than CK (Figure 7). It shows that these antioxidant enzymes, except POD, are more responsive towards NAC application and are mainly involved in alleviating the transplanting injury of rice seedlings. Besides SOD, which is involved in dismutating the most harmful superoxide into peroxides, both APX and GR are important components of the AsA-GSH cycle that are mainly involved in detoxifying excessive H_2_O_2_ in the plant cells [40]. Therefore, in the present study, the significantly higher activity of APX and GR could be linked with the enhanced AsA-GSH cycle in NAC plants because they suffered less cellular damage and showed significantly better growth than CK. A similar trend was noticed in non-enzymatic antioxidants, especially proline, which also verified that NAC priming improved the ROS-scavenging capacity of the rice seedlings to prevent transplanting injury (Figure 8). Taken together, these results indicate that NAC priming is a potential strategy to enhance the antioxidative capacity of plants under transplanting injury; the identification of main molecular drivers and detailed interplay between the enzymatic and non-enzymatic antioxidants could be a good subject for future studies.

### 3.4. The Mechanism of NAC Priming Mitigating the Transplanting Injury of Machine-Transplanted Rice

Transplanting injury causes oxidative damage to plants, which seriously affects the normal metabolic activities of plants. As an important energy production process in green plants, photosynthesis is very sensitive to stress. Studies have showed that the chlorophyll content and chlorophyll fluorescence parameters decreased under wound stress [35]. This study revealed that NAC priming increased chlorophyll content and net photosynthetic rate (Figure 4 and Figure 5), thereby promoting dry matter accumulation under transplanting injury (Figure 3). Therefore, NAC alleviated the effect of the transplanting injury on rice photosynthesis, which is beneficial in ensuring the energy required for various metabolic activities. In addition, treatments with NAC had a higher accumulation of antioxidants such as flavonoids and total phenols, which was beneficial for ROS scavenging (Figure 8 and Figure 9). However, the synthesis of excess antioxidants requires a lot of energy, which may cause growth stagnation [41]. Therefore, the improvement of photosynthesis by NAC also indirectly provides an energy basis for the antioxidant system. NAC enhances antioxidant enzyme activity (Figure 7) and reduces excessive membrane lipid peroxidation, protein denaturation, carbohydrate oxidation, chromolysis, and impairment of enzymatic activity [42]. This is conducive to the maintenance of enzyme activities related to metabolic activities such as photosynthesis and the stability of membrane structure. In conclusion, NAC improved the tolerance of machine-transplanted rice to transplanting injury by enhancing photosynthesis and antioxidant systems, and the improvement of any of these two systems also plays a positive role in the other system. NAC comprehensively regulates the tolerance of rice to the transplanting injury through the integration of these two systems.

## 4. Materials and Methods

### 4.1. Experiment Site

The experiment was conducted in the Danyang Experimental Base of Nanjing Agricultural University, Jiangsu Province, China (31°54′ N, 119°28′ E). The local conventional japonica rice NingJing 7 was used as the experimental variety. The experiment was carried out in plastic pots with a height of 35 cm and an inner diameter of 34 cm. Each pot was filled with 15 kg of soil up to 25 cm height, approximately. The experimental soil was taken from a local paddy, containing 1.12 g/kg nitrogen, 0.47 g/kg phosphorus, and 1.95 g/kg potassium.

### 4.2. Experimental Design

The seeds were soaked with different concentrations of NAC (CK, 0 μM; NAC 20, 20 μM; NAC 200, 200 μM) for 2 days. The volume ratio of the solution to the seed was 3:1. After soaking, the seeds were kept in the dark at 30 °C for 1 day. Then, the germinated seeds (120 g) were sown in the seedling tray (58 cm × 28 cm) containing organic substrates and cultivated for 20 days. When transplanted to pots, seedlings with consistent growth were selected to simulate mechanical transplanting by pruning roots down to 2 cm. Each pot contained three hills with three seedlings per hill. The rates of N, P_2_O_5_, and K_2_O per pot were 2.0 g, 1.0 g, and 1.6 g, respectively. N was applied split at the basal stage and panicle initiation stage; the ratio was 6:4. P and K were applied at the basal stage. Other management was consistent with the local conventional management. At the same time, the hydroponic experiments were carried out in order to visually capture the plant phenotype at 2 and 7 days after transplanting. The seedlings with pruned roots were fixed on the foam board. The nutrient solution was formulated according to Li et al. [43].

### 4.3. Parameter Measurements

Yield and yield components: At the maturity stage, three pots were selected by recording the average panicles to investigate yield components, such as panicles per pot, spikelets per panicle, grain-filling rate, and grain weight. Yield per pot = panicles × spikelets × grain-filling rate × grain weight. 

Tiller dynamics: Similar plants were selected for measurements of tiller dynamics in each barrel. The tillers of each plant were investigated every 7 days within 3 weeks after transplanting. Three repetitions were set for each treatment.

Plant height and dry weight: At 0, 2, and 7 days after transplanting, 10 similar plants were selected for each replicate. The plant height was measured from the base by using ruler. The plants were oven-dried at 105 °C for 30 min, followed by 80 °C to a constant weight. Three repetitions were set for each treatment.

Chloroplast pigment content: Fresh leaves (100 mg) crushed in liquid nitrogen were extracted in 10 mL of 95% ethanol [44]. Then, the extract was centrifuged at 10,000× *g* for 10 min. The absorbance at 663, 645, and 470 nm was measured with a UV–visible spectrophotometer. The chlorophyll and carotenoids contents were calculated as follows: 

Chlorophyll = 7.18 × OD663 + 17.32 × OD645; 

Carotenoids = 4.37 × OD470 + 2.10 × OD663 + 9.10 × OD645

Gas exchange parameters: Net photosynthetic rate (Pn), stomatal conductance (Gs), inter-cellular CO_2_ concentration (Ci), and transpiration rate (Tr) were measured by using an automatic portable photosynthesis Instrument (LC Pro-SD, Analytic Development Co., Ltd., Hoddesdon, UK) at 7.00 to 11.00 h on a sunny day. 

Sucrose content: Sucrose content was determined according to the method described by Hendrix [45]. The dry sample (100 mg) was extracted with 80% ethanol. The extract was mixed with 2M NaOH and boiled, then 30% HCl and 0.1% resorcinol were added, and the absorbance at 480 nm was measured with the UV–visible spectrophotometer. 

Antioxidant enzyme activity: Fresh leaves (0.5 g) were ground with 5 mL of extraction solution containing 0.2 mM ethylenediaminetetraacetic acid, 25 mM Hepes, 2 mM ascorbate, and polyvinylpyrrolidone (2%, *w/v*). After the homogenate was centrifuged (14,000× *g*) at 4 °C for 15 min, the supernatant was used to determine the activities of antioxidant enzymes. The corresponding reaction mixture solutions were prepared for measuring the activity of ascorbate peroxidase (APX), glutathione reductase (GR), catalase (CAT), peroxidase (POD), and superoxide dismutase (SOD) by following the method elaborated by Chen et al. [46].

The flavonoid and total phenol content: Fresh leaves (0.5 g) were homogenized with 5 mL of 70% (*v/v*) cold methanol containing 28% (*v/v*) ethanol and 2% (*v/v*) formic acid at room temperature for 2 h. Then, the homogenate was centrifuged (8000× *g*) for 15 min at 4 °C, and the supernatant was filtered through a 0.45 μm filter. The flavonoid content was determined according to Jia et al. [47], and the total phenol content was determined according to Djeridane et al. [48]. 

Proline content: 0.2 g of the crushed sample was added to 3 mL of 3% (*w/v*) sulfosalicylic acid and centrifuged at 3000× *g* for 20 min; then, the supernatant was mixed with 3 mL of hydrochloric acid and 1.5 mL of 2.5% ninhydrin. This mixture was boiled in a water bath for 1 h, cooled in an ice bath for 15 min, and extracted in 5 mL of toluene. After standing still, toluene was absorbed in the upper layer, and the absorbance value was measured at 520 nm [49].

H_2_O_2_ content: 0.1 g of leaf samples was homogenized in 5 mL of phosphate buffer (50 mM) with 1% (*w/v*) insoluble polyvinylpolypyrrolidone (PVPP). The extract was centrifuged (16,000× *g*) at 4 °C for 20 min [50]. The H_2_O_2_ content was estimated according to the instructions of the hydrogen peroxide assay kit (A064-1-1, Nanjing Jiancheng Bioengineering Institute, Nanjing, Jiangsu, China). 

MDA was determined following the method reported by Chen et al. [46]. The fresh leaves (0.1 g) were extracted in thiobarbituric acid solution. Then, the mixture was centrifuged (5000× *g*) at 25 °C for 10 min. The absorbance of supernatant was measured at 450 nm, 532 nm, and 600 nm by using a UV–visible spectrophotometer. The MDA content was calculated as follows:

(6.45 × (OD535 − OD600) − 0.56 × OD450) × V/W

V: extract volume (mL), W: fresh weight (g)

### 4.4. Statistic Analysis

All the data were analyzed by SPSS 18.0 and are presented as means ± SD (*n* = 3). Data were analyzed by ANOVA and the mean of treatments were compared by Duncan’s Multiple Range Test (*p* < 0.05).

## 5. Conclusions

In conclusion, the results presented in the study showed that seed priming with NAC could be adopted as a promising strategy to alleviate the initial transplanting shock on rice seedlings. We noticed that seed priming with 20 µM NAC is more effective in promoting the growth and yield of rice seedlings; however, comparative studies in the future with a greater number of concentrations could help to explore the underlying molecular mechanisms and detailed response of rice seedlings in terms of redox homeostasis and yield components. In the present study, the better growth of NAC-treated seedlings was mainly attributed to better ROS-scavenging capacity than CK, which protected the rice seedlings from cellular damage during initial days of transplanting. Consequently, these seedlings retained higher pigment content and net photosynthesis because they accumulated more sucrose and dry matter, and had significantly higher tillering and yield than CK plants. The results in the present study provide a direction for future studies to explore the detailed role of exogenous NAC in improving the stress tolerance and yield of rice crops.

## Figures and Tables

**Table 1:** Effects of different concentrations of NAC on yield and yield components.

Treatment	Panicles Pot^−1^	Spikelets Panicle^−1^	Grain Filling (%)	Grain Weight (mg)	Grain Yield (g·pot^−1^)
CK	28.7 ± 1.2 b	153.3 ± 5.5 a	90.2 ± 1.5 b	28.5 ± 0.2 a	112.6 ± 2.2 b
NAC-20	31.4 ± 1.0 a	149.1 ± 6.6 a	92.9 ± 0.7 a	28.8 ± 0.4 a	125.2 ± 2.7 a
NAC-200	30.9 ± 0.7 ab	148.4 ± 5.2 a	92.6 ± 0.9 ab	28.7 ± 0.5 a	121.7 ± 4.6 a

CK, 0 μm; NAC-20, 20 μm; NAC-200, 200 μm. Data are means ± SD (*n* = 3). The different letters following average values indicate significant difference (Duncan, *p* < 0.05).
